# Crystal structure of *trans*-di­aqua­bis­(nicotinamide-κ*N* ^1^)bis­(4-nitro­benzoato-κ*O*)manganese(II)

**DOI:** 10.1107/S2056989016005612

**Published:** 2016-04-08

**Authors:** Gülçin Şefiye Aşkın, Hacali Necefoğlu, Ali Murat Tonbul, Nefise Dilek, Tuncer Hökelek

**Affiliations:** a Department of Physics, Hacettepe University, 06800 Beytepe, Ankara, Turkey; b Department of Chemistry, Kafkas University, 36100 Kars, Turkey; c International Scientific Research Centre, Baku State University, 1148 Baku, Azerbaijan; d Department of Physics, Aksaray University, 68100, Aksaray, Turkey

**Keywords:** crystal structure, manganese(II), nicotinamide, 4-nitro­benzoic acid, transition metal complex

## Abstract

The asymmetric unit of the title compound, [Mn(C_7_H_4_NO_4_)_2_(C_6_H_6_N_2_O)_2_(H_2_O)_2_], contains one Mn^II^ atom, one 4-nitro­benzoate (NB) anion, one nicotinamide (NA) ligand and one water mol­ecule; NA and NB each act as a monodentate ligand. The Mn^II^ atom, lying on an inversion centre, is coordinated by four O atoms and two pyridine N atoms in a distorted octa­hedral geometry. The water mol­ecules are hydrogen bonded to the carboxyl­ate O atoms. The dihedral angle between the carboxyl­ate group and the adjacent benzene ring is 24.4 (3)°, while the benzene and pyridine rings are oriented at a dihedral angle of 86.63 (11)°. In the crystal, O—H⋯O and N—H⋯O hydrogen bonds link the mol­ecules, forming a layer parallel to the *ab* plane. The layers are further linked *via* weak C—H⋯O hydrogen bonds, a π–π stacking inter­action [centroid–centroid distance = 3.868 (2) Å] and a weak C—H⋯π inter­action, resulting in a three-dimensional network.

## Abstract 2

In the title centrosymmetric Mn^II^ complex, the Mn^II^ atom is coordinated by two 4-nitro­benzoate (NB) anions, two nicotinamide (NA) ligands and two water mol­ecules; the NB and NA ligands act as monodentate ligands. The resulting MnN_2_O_4_ coordination polyhedron is a distorted octa­hedron.

## Chemical context   

Nicotinamide (NA) is one form of niacin. A deficiency of this vitamin leads to loss of copper from the body, known as pellagra disease. The NA ring is the reactive part of nicotinamide adenine dinucleotide (NAD) and its phosphate (NADP), which are the major electron carriers in many biological oxidation–reduction reactions (You *et al.*, 1978 ▸). The nicotinic acid derivative *N*,*N*-di­ethyl­nicotinamide (DENA) is an important respiratory stimulant (Bigoli *et al.*, 1972 ▸). Transition metal complexes with biochemical mol­ecules show inter­esting physical and/or chemical properties with potential applications in biological systems (Antolini *et al.*, 1982 ▸).
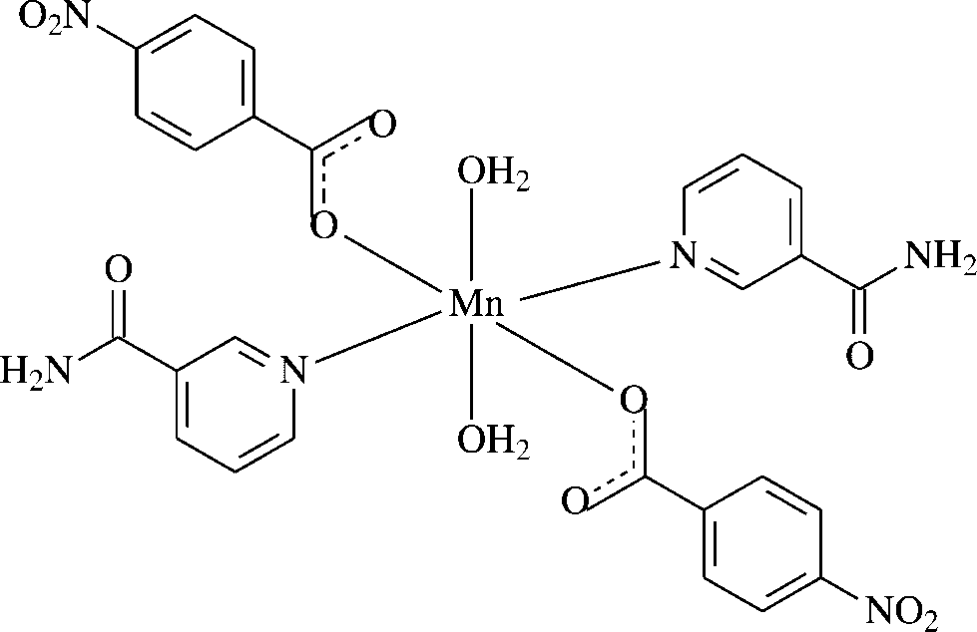


Crystal structures of metal complexes with benzoic acid derivatives have been reported extensively because of the varieties of their coordination modes. For example, Co and Cd complexes with 4-amino­benzoic acid (Chen & Chen, 2002 ▸; Amiraslanov *et al.*, 1979 ▸; Hauptmann *et al.*, 2000 ▸), Co complexes with benzoic acid (Catterick *et al.*, 1974 ▸), 4-nitro­benzoic acid (Nadzhafov *et al.*, 1981 ▸) and phthalic acid (Adiwidjaja *et al.*, 1978 ▸), and Cu with 4-hy­droxy­benzoic acid (Shnulin *et al.*, 1981 ▸) have been described. Mn complexes closely related to the title compound, di­aqua­bis­(4-nitro­benzoato)bis­(1*H*-1,2,4-triazol-3-amine)­manganese (Zhang *et al.*, 2013 ▸) and di­aqua­bis­(1*H*-imidazole)­bis­(4-nitro­benzoato)manganese (Xu & Xu, 2004 ▸), have also been reported.

## Structural commentary   

**Figure 1:**
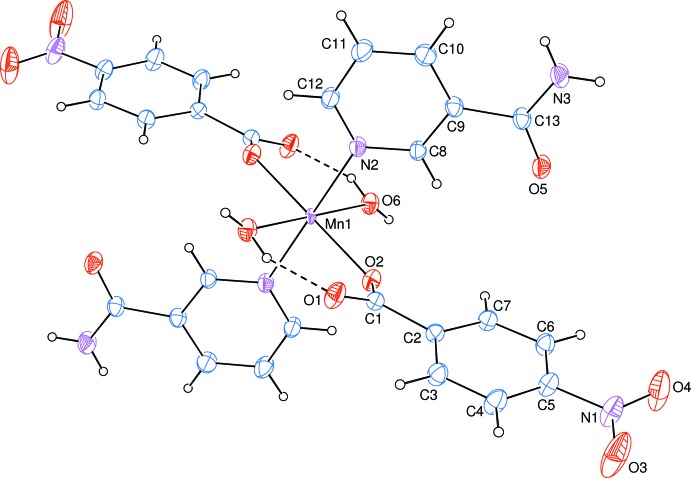
The mol­ecular structure of the title compound with the atom-numbering scheme. Displacement ellipsoids are drawn at the 40% probability level. Intra­molecular O—H⋯O hydrogen bonds are shown as dashed lines. Unlabelled atoms are symmetry-related to labelled atoms by (−*x*, −*y*, −*z*).

The asymmetric unit of the title mononuclear complex contains one Mn^II^ atom (site symmetry 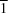), one 4-nitro­benzoate (NB) anion, one nicotinamide (NA) ligand and one water mol­ecule, all ligands coordinating in a monodentate manner. In the complex, the two carboxyl­ate O atoms (O2 and O2^iii^) of the two symmetry-related monodentate NB anions and the two symmetry-related water O atoms (O6 and O6^iii^) around the Mn^II^ atom form a slightly distorted square-planar arrangement, while the slightly distorted octa­hedral coordination sphere is completed by the two pyridine N atoms (N2 and N2^iii^) of the two symmetry-related monodentate NA ligands in the axial positions [symmetry code: (iii) −*x*, −*y*, −*z*; Fig. 1 ▸].

The near equality of C—O bond lengths [C1—O1 = 1.253 (4) and C1—O2 = 1.248 (4) Å] in the carboxyl­ate group indicates delocalized bonds rather than localized single and double bonds. The Mn—O bond lengths [2.156 (2) and 2.115 (2) Å] and the Mn—N bond length [2.134 (3) Å] are close to the standard values. Atom Mn1 lies 0.4172 (1) Å above the O1/O2/C1 plane of the carboxyl­ate group. The O—Mn—O and O—Mn—N bond angles deviate slightly from the ideal value of 90°. The dihedral angle between the carboxyl­ate group (O1/O2/C1) and the adjacent benzene (C2–C7) ring is 24.4 (3)°, while the benzene ring and the pyridine (N2/C8–C12) ring are oriented at a dihedral angle of 86.63 (11)°.

## Supra­molecular features   

**Figure 2:**
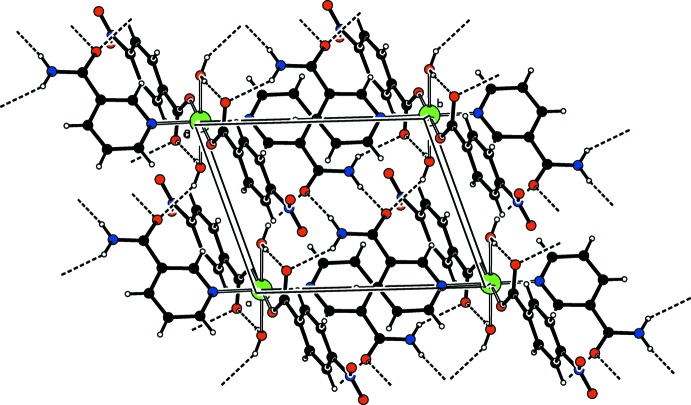
A packing diagram of the title compound, viewed down the *c* axis. Inter­molecular O—H⋯O and N—H⋯O hydrogen bonds are shown as dashed lines.

In the crystal, inter­molecular N—H_na_⋯O_na_ (na = nicotinamide), N—H_na_⋯O_c_ (c = carboxyl­ate group) and O—H_w_⋯O_na_ (w = water) hydrogen bonds (Table 1 ▸) link the mol­ecules, forming a layer parallel to the *ab* plane (Fig. 2 ▸). In the layer, 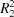
(8) and 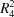
(8) ring motifs are observed. The layers are further linked *via* weak C—H⋯O hydrogen bonds, a weak C—H⋯π inter­action (Table 1 ▸) and a π–π inter­action between the benzene rings [*Cg*1⋯*Cg*1^ix^ = 3.868 (2) Å; symmetry code: (ix) 1 − *x*, −*y*, 1 − *z*, where *Cg*1 is the centroid of the C2–C7 ring].

## Synthesis and crystallization   

The title compound was prepared by the reaction of MnSO_4_·H_2_O (0.85 g, 25 mmol) in H_2_O (25 ml) and nicotinamide (1.22 g, 10 mmol) in H_2_O (25 ml) with sodium 4-nitro­benzoate (1.90 g, 10 mmol) in H_2_O (150 ml). The mixture was filtered and set aside to crystallize at ambient temperature for one week, giving colourless single crystals.

## Refinement   

The experimental details including the crystal data, data collection and refinement are summarized in Table 2 ▸. Atoms H61 and H62 of the water mol­ecule and atoms H3*A* and H3*B* of the NH_2_ group were located in a difference Fourier map, and their coordinates were refined with distance restraints of O—H = 0.85 (2) Å and N—H = 0.86 (2) Å, and with *U*_iso_(H) = 1.5*U*_eq_(O,N). The C-bound H atoms were positioned geometrically with C—H = 0.93 Å and were constrained to ride on their parent atoms with *U*_iso_(H) = 1.2*U*_eq_(C).

## Supplementary Material

Crystal structure: contains datablock(s) I, global. DOI: 10.1107/S2056989016005612/is5449sup1.cif

Structure factors: contains datablock(s) I. DOI: 10.1107/S2056989016005612/is5449Isup2.hkl

CCDC reference: 1472331

Additional supporting information:  crystallographic information; 3D view; checkCIF report

## Figures and Tables

**Table 1:** Hydrogen-bond geometry (Å, °) *Cg*2 is the centroid of the N2/C8–C12 ring.

*D*—H⋯*A*	*D*—H	H⋯*A*	*D*⋯*A*	*D*—H⋯*A*
N3—H3*A*⋯O5^i^	0.90 (5)	2.07 (6)	2.898 (6)	152 (4)
N3—H3*B*⋯O1^ii^	0.90 (4)	2.19 (5)	2.923 (4)	138 (4)
O6—H61⋯O1^iii^	0.90 (4)	1.78 (5)	2.646 (5)	161 (4)
O6—H62⋯O5^iv^	0.89 (3)	2.10 (4)	2.897 (3)	148 (4)
C3—H3⋯O4^v^	0.93	2.59	3.456 (6)	156
C6—H6⋯O1^vi^	0.93	2.40	3.319 (4)	170
C12—H12⋯O3^vii^	0.93	2.54	3.416 (7)	157
C4—H4⋯*Cg*2^viii^	0.93	2.91	3.827 (4)	172

Symmetry codes: (i) 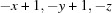; (ii) 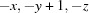; (iii) 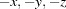; (iv) 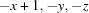; (v) 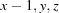; (vi) 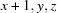; (vii) 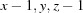; (viii) 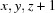.

**Table 2:** Experimental details

Crystal data	
Chemical formula	[Mn(C_7_H_4_NO_4_)_2_(C_6_H_6_N_2_O)_2_(H_2_O)_2_]
*M*_r_	667.45
Crystal system, space group	Triclinic, *P* 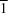
Temperature (K)	296
*a*, *b*, *c* (Å)	7.6051 (3), 10.0027 (4), 10.2152 (4)
α, β, γ (°)	78.067 (3), 88.430 (4), 71.746 (3)
*V* (Å^3^)	721.45 (5)
*Z*	1
Radiation type	Mo *K*α
μ (mm^−1^)	0.53
Crystal size (mm)	0.45 × 0.35 × 0.32

Data collection	
Diffractometer	Bruker SMART BREEZE CCD
Absorption correction	Multi-scan (*SADABS*; Bruker, 2012 ▸)
*T*_min_, *T*_max_	0.765, 0.815
No. of measured, independent and observed [*I* > 2σ(*I*)] reflections	17255, 3595, 3475
*R*_int_	0.027
(sin θ/λ)_max_ (Å^−1^)	0.669

Refinement	
*R*[*F* ^2^ > 2σ(*F* ^2^)], *wR*(*F* ^2^), *S*	0.058, 0.178, 1.17
No. of reflections	3595
No. of parameters	217
No. of restraints	4
H-atom treatment	H atoms treated by a mixture of independent and constrained refinement
Δρ_max_, Δρ_min_ (e Å^−3^)	1.00, −0.50

Computer programs: *APEX2* and *SAINT* (Bruker, 2012 ▸), *SHELXS97* and *SHELXL97* (Sheldrick, 2008 ▸), *ORTEP-3 for Windows* and *WinGX* (Farrugia, 2012 ▸) and *PLATON* (Spek, 2009 ▸).

## supplementary crystallographic information

### Crystal data

**Table:**

[Mn(C_7_H_4_NO_4_)_2_(C_6_H_6_N_2_O)_2_(H_2_O)_2_]	*Z* = 1
*M**_r_* = 667.45	*F*(000) = 343
Triclinic, *P*1	*D*_x_ = 1.536 Mg m^−^^3^
Hall symbol: -P 1	Mo *K*α radiation, λ = 0.71073 Å
*a* = 7.6051 (3) Å	Cell parameters from 9891 reflections
*b* = 10.0027 (4) Å	θ = 2.2–28.4°
*c* = 10.2152 (4) Å	µ = 0.53 mm^−^^1^
α = 78.067 (3)°	*T* = 296 K
β = 88.430 (4)°	Prism, colourless
γ = 71.746 (3)°	0.45 × 0.35 × 0.32 mm
*V* = 721.45 (5) Å^3^	

### Data collection

**Table:**

Bruker SMART BREEZE CCD diffractometer	3595 independent reflections
Radiation source: fine-focus sealed tube	3475 reflections with *I* > 2σ(*I*)
Graphite monochromator	*R*_int_ = 0.027
φ and ω scans	θ_max_ = 28.4°, θ_min_ = 2.0°
Absorption correction: multi-scan (*SADABS*; Bruker, 2012)	*h* = −10→9
*T*_min_ = 0.765, *T*_max_ = 0.815	*k* = −12→13
17255 measured reflections	*l* = −13→13

### Refinement

**Table:**

Refinement on *F*^2^	Primary atom site location: structure-invariant direct methods
Least-squares matrix: full	Secondary atom site location: difference Fourier map
*R*[*F*^2^ > 2σ(*F*^2^)] = 0.058	Hydrogen site location: inferred from neighbouring sites
*wR*(*F*^2^) = 0.178	H atoms treated by a mixture of independent and constrained refinement
*S* = 1.17	*w* = 1/[σ^2^(*F*_o_^2^) + (0.0908*P*)^2^ + 0.7889*P*] where *P* = (*F*_o_^2^ + 2*F*_c_^2^)/3
3595 reflections	(Δ/σ)_max_ < 0.001
217 parameters	Δρ_max_ = 1.00 e Å^−^^3^
4 restraints	Δρ_min_ = −0.50 e Å^−^^3^

### Special details

**Table:**

Geometry. All esds (except the esd in the dihedral angle between two l.s. planes) are estimated using the full covariance matrix. The cell esds are taken into account individually in the estimation of esds in distances, angles and torsion angles; correlations between esds in cell parameters are only used when they are defined by crystal symmetry. An approximate (isotropic) treatment of cell esds is used for estimating esds involving l.s. planes.
Refinement. Refinement of F^2^ against ALL reflections. The weighted R-factor wR and goodness of fit S are based on F^2^, conventional R-factors R are based on F, with F set to zero for negative F^2^. The threshold expression of F^2^ > 2sigma(F^2^) is used only for calculating R-factors(gt) etc. and is not relevant to the choice of reflections for refinement. R-factors based on F^2^ are statistically about twice as large as those based on F, and R- factors based on ALL data will be even larger.

### Fractional atomic coordinates and isotropic or equivalent isotropic displacement parameters (Å^2^)

**Table:**

	*x*	*y*	*z*	*U*_iso_*/*U*_eq_	
Mn1	0.0000	0.0000	0.0000	0.02437 (17)	
O1	−0.1121 (4)	0.1452 (4)	0.2673 (3)	0.0638 (7)	
O2	0.1322 (3)	0.0165 (3)	0.1728 (2)	0.0479 (5)	
O3	0.4619 (6)	0.2860 (8)	0.6872 (5)	0.147 (3)	
O4	0.6545 (6)	0.2824 (7)	0.5355 (5)	0.124 (2)	
O5	0.4269 (4)	0.3417 (3)	−0.0003 (3)	0.0598 (7)	
O6	0.2722 (3)	−0.0746 (3)	−0.0769 (3)	0.0506 (6)	
H61	0.227 (7)	−0.084 (6)	−0.154 (3)	0.076*	
H62	0.377 (4)	−0.135 (4)	−0.034 (5)	0.076*	
N1	0.5113 (5)	0.2645 (5)	0.5801 (4)	0.0736 (11)	
N2	−0.0042 (4)	0.2124 (3)	−0.0942 (3)	0.0427 (6)	
N3	0.3202 (5)	0.5611 (4)	−0.1340 (4)	0.0645 (9)	
H3A	0.424 (5)	0.579 (6)	−0.113 (6)	0.097*	
H3B	0.227 (6)	0.623 (5)	−0.189 (5)	0.097*	
C1	0.0585 (4)	0.0898 (3)	0.2563 (3)	0.0409 (6)	
C2	0.1853 (4)	0.1239 (3)	0.3472 (3)	0.0406 (6)	
C3	0.1181 (5)	0.1682 (5)	0.4643 (4)	0.0533 (8)	
H3	−0.0002	0.1685	0.4902	0.064*	
C4	0.2265 (5)	0.2118 (5)	0.5428 (4)	0.0605 (10)	
H4	0.1835	0.2402	0.6220	0.073*	
C5	0.3994 (5)	0.2121 (4)	0.5000 (4)	0.0516 (8)	
C6	0.4712 (5)	0.1680 (4)	0.3862 (3)	0.0489 (7)	
H6	0.5888	0.1696	0.3601	0.059*	
C7	0.3619 (5)	0.1205 (4)	0.3104 (3)	0.0455 (7)	
H7	0.4088	0.0863	0.2344	0.055*	
C8	0.1370 (4)	0.2581 (3)	−0.0689 (3)	0.0417 (6)	
H8	0.2343	0.1949	−0.0119	0.050*	
C9	0.1454 (4)	0.3939 (3)	−0.1231 (3)	0.0428 (6)	
C10	−0.0002 (6)	0.4870 (4)	−0.2088 (4)	0.0617 (10)	
H10	0.0003	0.5796	−0.2475	0.074*	
C11	−0.1449 (6)	0.4407 (4)	−0.2358 (5)	0.0666 (11)	
H11	−0.2433	0.5015	−0.2932	0.080*	
C12	−0.1424 (5)	0.3030 (4)	−0.1765 (4)	0.0501 (7)	
H12	−0.2409	0.2724	−0.1948	0.060*	
C13	0.3087 (5)	0.4309 (3)	−0.0821 (4)	0.0464 (7)	

### Atomic displacement parameters (Å^2^)

**Table:**

	*U*^11^	*U*^22^	*U*^33^	*U*^12^	*U*^13^	*U*^23^
Mn1	0.0212 (3)	0.0226 (3)	0.0332 (3)	−0.01052 (18)	−0.00068 (17)	−0.00816 (18)
O1	0.0382 (12)	0.091 (2)	0.0654 (16)	−0.0140 (13)	−0.0016 (11)	−0.0323 (15)
O2	0.0433 (12)	0.0484 (12)	0.0548 (13)	−0.0132 (9)	−0.0050 (9)	−0.0175 (10)
O3	0.081 (3)	0.287 (7)	0.131 (4)	−0.070 (4)	0.026 (3)	−0.155 (5)
O4	0.087 (3)	0.220 (6)	0.126 (4)	−0.093 (3)	0.027 (3)	−0.102 (4)
O5	0.0565 (15)	0.0446 (12)	0.0814 (18)	−0.0224 (11)	−0.0182 (13)	−0.0067 (12)
O6	0.0376 (11)	0.0527 (13)	0.0614 (14)	−0.0119 (10)	0.0006 (10)	−0.0152 (11)
N1	0.0497 (18)	0.104 (3)	0.079 (2)	−0.0200 (19)	−0.0025 (16)	−0.052 (2)
N2	0.0390 (12)	0.0402 (12)	0.0517 (14)	−0.0156 (10)	−0.0025 (10)	−0.0104 (11)
N3	0.067 (2)	0.0450 (16)	0.087 (2)	−0.0305 (15)	−0.0144 (18)	−0.0052 (15)
C1	0.0398 (14)	0.0415 (14)	0.0414 (14)	−0.0142 (12)	−0.0017 (11)	−0.0062 (11)
C2	0.0383 (14)	0.0410 (14)	0.0411 (14)	−0.0117 (11)	−0.0015 (11)	−0.0066 (11)
C3	0.0396 (15)	0.074 (2)	0.0493 (17)	−0.0174 (15)	0.0056 (13)	−0.0201 (16)
C4	0.0453 (18)	0.092 (3)	0.0505 (18)	−0.0181 (18)	0.0062 (14)	−0.0341 (19)
C5	0.0430 (16)	0.061 (2)	0.0534 (18)	−0.0133 (14)	−0.0036 (13)	−0.0231 (15)
C6	0.0385 (15)	0.0613 (19)	0.0509 (17)	−0.0173 (14)	0.0034 (13)	−0.0179 (15)
C7	0.0420 (15)	0.0530 (17)	0.0441 (15)	−0.0149 (13)	0.0043 (12)	−0.0162 (13)
C8	0.0394 (14)	0.0385 (14)	0.0499 (16)	−0.0154 (11)	−0.0037 (12)	−0.0091 (12)
C9	0.0440 (15)	0.0358 (13)	0.0514 (16)	−0.0144 (12)	0.0007 (12)	−0.0124 (12)
C10	0.065 (2)	0.0387 (16)	0.078 (3)	−0.0181 (16)	−0.0145 (19)	0.0012 (16)
C11	0.057 (2)	0.0495 (19)	0.085 (3)	−0.0138 (16)	−0.027 (2)	0.0046 (18)
C12	0.0424 (16)	0.0481 (17)	0.0606 (19)	−0.0155 (13)	−0.0095 (14)	−0.0097 (14)
C13	0.0467 (16)	0.0386 (14)	0.0586 (18)	−0.0171 (12)	0.0001 (13)	−0.0144 (13)

### Geometric parameters (Å, º)

**Table:**

Mn1—O2	2.115 (2)	C2—C7	1.377 (4)
Mn1—O2^i^	2.115 (2)	C3—C4	1.385 (5)
Mn1—O6	2.156 (2)	C3—H3	0.9300
Mn1—O6^i^	2.156 (2)	C4—H4	0.9300
Mn1—N2	2.134 (3)	C5—N1	1.471 (5)
Mn1—N2^i^	2.134 (3)	C5—C4	1.375 (5)
O1—C1	1.253 (4)	C6—C5	1.368 (5)
O2—C1	1.248 (4)	C6—C7	1.397 (5)
O3—N1	1.187 (5)	C6—H6	0.9300
O4—N1	1.219 (5)	C7—H7	0.9300
O5—C13	1.238 (4)	C8—C9	1.377 (4)
O6—H61	0.903 (19)	C8—H8	0.9300
O6—H62	0.892 (19)	C9—C10	1.389 (5)
N2—C8	1.342 (4)	C9—C13	1.495 (4)
N2—C12	1.330 (4)	C10—C11	1.375 (6)
N3—C13	1.330 (4)	C10—H10	0.9300
N3—H3A	0.90 (2)	C11—H11	0.9300
N3—H3B	0.90 (2)	C12—C11	1.380 (5)
C2—C1	1.515 (4)	C12—H12	0.9300
C2—C3	1.390 (4)		
			
O2^i^—Mn1—O2	180.00 (7)	C2—C3—H3	119.8
O2—Mn1—O6	87.19 (10)	C4—C3—C2	120.3 (3)
O2^i^—Mn1—O6	92.81 (10)	C4—C3—H3	119.8
O2—Mn1—O6^i^	92.81 (10)	C3—C4—H4	121.0
O2^i^—Mn1—O6^i^	87.19 (10)	C5—C4—C3	118.1 (3)
O2—Mn1—N2	89.98 (10)	C5—C4—H4	121.0
O2^i^—Mn1—N2	90.02 (10)	C4—C5—N1	118.1 (3)
O2—Mn1—N2^i^	90.02 (10)	C6—C5—N1	118.5 (3)
O2^i^—Mn1—N2^i^	89.98 (10)	C6—C5—C4	123.4 (3)
O6—Mn1—O6^i^	180.00 (13)	C5—C6—C7	117.7 (3)
N2—Mn1—O6	87.00 (10)	C5—C6—H6	121.1
N2^i^—Mn1—O6	93.00 (10)	C7—C6—H6	121.1
N2—Mn1—O6^i^	93.00 (10)	C2—C7—C6	120.6 (3)
N2^i^—Mn1—O6^i^	87.00 (10)	C2—C7—H7	119.7
N2^i^—Mn1—N2	180.00 (7)	C6—C7—H7	119.7
C1—O2—Mn1	126.2 (2)	N2—C8—C9	123.4 (3)
Mn1—O6—H61	93 (3)	N2—C8—H8	118.3
Mn1—O6—H62	129 (3)	C9—C8—H8	118.3
H61—O6—H62	124 (5)	C8—C9—C10	117.7 (3)
O3—N1—O4	121.7 (4)	C8—C9—C13	117.2 (3)
O3—N1—C5	119.3 (4)	C10—C9—C13	125.1 (3)
O4—N1—C5	119.0 (4)	C9—C10—H10	120.4
C8—N2—Mn1	119.1 (2)	C11—C10—C9	119.3 (3)
C12—N2—Mn1	122.8 (2)	C11—C10—H10	120.4
C12—N2—C8	118.1 (3)	C10—C11—C12	119.1 (3)
C13—N3—H3A	117 (4)	C10—C11—H11	120.4
C13—N3—H3B	118 (4)	C12—C11—H11	120.4
H3A—N3—H3B	125 (5)	N2—C12—C11	122.4 (3)
O1—C1—C2	116.4 (3)	N2—C12—H12	118.8
O2—C1—O1	126.0 (3)	C11—C12—H12	118.8
O2—C1—C2	117.5 (3)	O5—C13—N3	122.0 (3)
C3—C2—C1	119.5 (3)	O5—C13—C9	119.8 (3)
C7—C2—C1	120.5 (3)	N3—C13—C9	118.2 (3)
C7—C2—C3	119.9 (3)		
			
O6—Mn1—O2—C1	160.3 (3)	C7—C2—C3—C4	1.4 (6)
O6^i^—Mn1—O2—C1	−19.7 (3)	C1—C2—C7—C6	172.5 (3)
N2—Mn1—O2—C1	73.3 (3)	C3—C2—C7—C6	−3.2 (5)
N2^i^—Mn1—O2—C1	−106.7 (3)	C2—C3—C4—C5	1.0 (6)
O2—Mn1—N2—C8	34.5 (2)	C4—C5—N1—O3	10.2 (8)
O2^i^—Mn1—N2—C8	−145.5 (2)	C4—C5—N1—O4	−171.0 (5)
O2—Mn1—N2—C12	−144.8 (3)	C6—C5—N1—O3	−170.6 (6)
O2^i^—Mn1—N2—C12	35.2 (3)	C6—C5—N1—O4	8.1 (7)
O6—Mn1—N2—C8	−52.7 (2)	N1—C5—C4—C3	177.4 (4)
O6^i^—Mn1—N2—C8	127.3 (2)	C6—C5—C4—C3	−1.8 (7)
O6—Mn1—N2—C12	128.0 (3)	C7—C6—C5—N1	−179.1 (4)
O6^i^—Mn1—N2—C12	−52.0 (3)	C7—C6—C5—C4	0.0 (6)
Mn1—O2—C1—O1	14.1 (5)	C5—C6—C7—C2	2.5 (5)
Mn1—O2—C1—C2	−161.6 (2)	N2—C8—C9—C10	−0.4 (5)
Mn1—N2—C8—C9	−178.9 (2)	N2—C8—C9—C13	178.0 (3)
C12—N2—C8—C9	0.4 (5)	C8—C9—C10—C11	0.1 (6)
Mn1—N2—C12—C11	179.2 (3)	C13—C9—C10—C11	−178.3 (4)
C8—N2—C12—C11	−0.1 (6)	C8—C9—C13—O5	−1.5 (5)
C3—C2—C1—O1	22.0 (5)	C8—C9—C13—N3	179.5 (3)
C3—C2—C1—O2	−161.8 (3)	C10—C9—C13—O5	176.8 (4)
C7—C2—C1—O1	−153.7 (3)	C10—C9—C13—N3	−2.2 (6)
C7—C2—C1—O2	22.4 (4)	C9—C10—C11—C12	0.3 (7)
C1—C2—C3—C4	−174.4 (4)	N2—C12—C11—C10	−0.3 (7)

Symmetry code: (i) −*x*, −*y*, −*z*.

### Hydrogen-bond geometry (Å, º)

*Cg*2 is the centroid of the N2/C8–C12 ring.

**Table:**

*D*—H···*A*	*D*—H	H···*A*	*D*···*A*	*D*—H···*A*
N3—H3*A*···O5^ii^	0.90 (5)	2.07 (6)	2.898 (6)	152 (4)
N3—H3*B*···O1^iii^	0.90 (4)	2.19 (5)	2.923 (4)	138 (4)
O6—H61···O1^i^	0.90 (4)	1.78 (5)	2.646 (5)	161 (4)
O6—H62···O5^iv^	0.89 (3)	2.10 (4)	2.897 (3)	148 (4)
C3—H3···O4^v^	0.93	2.59	3.456 (6)	156
C6—H6···O1^vi^	0.93	2.40	3.319 (4)	170
C12—H12···O3^vii^	0.93	2.54	3.416 (7)	157
C4—H4···*Cg*2^viii^	0.93	2.91	3.827 (4)	172

Symmetry codes: (i) −*x*, −*y*, −*z*; (ii) −*x*+1, −*y*+1, −*z*; (iii) −*x*, −*y*+1, −*z*; (iv) −*x*+1, −*y*, −*z*; (v) *x*−1, *y*, *z*; (vi) *x*+1, *y*, *z*; (vii) *x*−1, *y*, *z*−1; (viii) *x*, *y*, *z*+1.
